# Maternal smoking during pregnancy and risk factors for cardiovascular disease in adulthood

**DOI:** 10.1016/j.atherosclerosis.2011.08.018

**Published:** 2011-12

**Authors:** Bernardo Lessa Horta, Denise P. Gigante, Aydin Nazmi, Vera Maria F. Silveira, Isabel Oliveira, Cesar G. Victora

**Affiliations:** a Post-Graduate Programme in Epidemiology, Universidade Federal de Pelotas, Rua Marechal Deodora 1160 – 3 andar, 96090-790 Pelotas, RS, Brazil; b Food Science and Nutrition, California Polytechnic State University, 1 Grand Avenue, San Luis Obispo, CA 93407, USA

**Keywords:** Maternal smoking, Pregnancy, Cardiovascular, Risk factor

## Abstract

**Objective:**

This study was aimed at assessing the effect of maternal smoking during pregnancy on metabolic cardiovascular risk factors in early adulthood in a Brazilian birth cohort, after controlling for possible confounding variables and health behaviors in early adulthood.

**Methods:**

In 1982, the maternity hospitals in Pelotas, southern Brazil, were visited and all births were identified. Those livebirths whose family lived in the urban area of the city were studied prospectively. In 2004–2005, we attempted to follow the whole cohort, the subjects were interviewed, examined and blood sample was collected. The following outcomes were studied: blood pressure; HDL cholesterol; triglycerides; random blood glucose and C-reactive protein. To explore the effect of maternal smoking, we adjusted the coefficients for the following possible mediators: perinatal factors (low birthweight and preterm births); adult behavioral factors (physical activity, dietary pattern, intake of fat and fiber, and tobacco smoking) and adult anthropometry (body mass index and waist circumference).

**Results:**

In 2004–2005, we interviewed 4297 subjects, with a follow-up rate of 77.4%. The only significant finding in the unadjusted analyses was lower HDL cholesterol among females. After adjustment for lifestyle variables in early adulthood, birthweight and waist circumference, the difference in HDL levels between offspring of smokers and non-smokers reduced from −2.10 mg/dL (95% confidence interval: −3.39; −0.80) to −1.03 mg/dL (−2.35; 0.30).

**Conclusion:**

Evidence that maternal smoking during pregnancy programs offspring metabolic cardiovascular risk factors are scarce, and reported associations are likely due to postnatal exposure to lifestyle patterns.

## 1 Introduction

Smoking is the second leading risk factor for global mortality [1]. In addition, exposure to maternal smoking during pregnancy is related to short and long-term health risks, ranging from intrauterine growth restriction [2,3] to psychological problems. [4] However, the evidence concerning the programming of cardiovascular diseases by maternal smoking during pregnancy is not clear-cut. A meta-analysis that included nine studies reported a small increase in blood pressure (0.62 mmHg; 95% confidence interval: 0.19; 1.05) among subjects prenatally exposed to smoking. [5] With respect to blood lipids, Jaddoe et al. [6] observed that maternal smoking in pregnancy is associated with a rise in total cholesterol levels and a tendency towards an adverse lipoprotein profile, with a decrease in HDL and an increase in LDL levels. Power et al. [8] observed that offspring of smokers had higher triglyceride levels, whereas their HDL cholesterol was lower than for adults who had not been exposed in utero.

Evidence on long-term effects of smoking during pregnancy on the offspring is limited to observational studies, and therefore may be affected by selection bias and confounding. Several strategies were used in the literature to overcome this problem. Brion et al. [7] reported that the effects of paternal and maternal smoking on blood pressure at 7 years were similar, although one would expect a stronger association with maternal smoking, as the latter is more strongly related to intrauterine growth than environmental tobacco exposure resulting from paternal smoking. [3] These authors suggest that maternal smoking may be a marker for familial factors related to blood pressure, rather than a cause of fetal programming. Reinforcing such evidence, the associations described by Power et al. [8] vanished when the estimates were also adjusted for behavioral variables in adulthood, such as physical activity, diet and tobacco smoking, suggesting that the observed effect of maternal smoking in pregnancy on metabolic cardiovascular risk factors was mediated by healthy behaviors in adulthood and not due to a prenatal programming.

The present study was aimed at assessing the effect of maternal smoking in the pregnancy on metabolic cardiovascular risk factors in early adulthood in a Brazilian birth cohort followed up for 22–23 years, after controlling for possible confounding variables and health behaviors in early adulthood.

## 2 Methods

In 1982, the five maternity hospitals in Pelotas, a southern Brazilian city, were visited daily and all births were identified. The 5914 liveborns whose family lived in the urban area were examined and their mothers interviewed. These subjects have been followed-up for several times [9].

From October 2004 to August 2005, all households located in urban area of the city were visited in search of subjects born in 1982 and belonging to the cohort. For those who had not been located and were not known to have died, we used the last known address and existing databases (including universities, secondary schools and telephone directories) for another attempt. Subjects answered a questionnaire and were examined. At the end of the interview, the subjects were invited to visit the research laboratory to donate a blood sample, collected by venous puncture.

The following metabolic risk factors of cardiovascular diseases were assessed:

• Blood pressure: blood pressure was measured at the beginning and at the end of the interview, using a digital wrist sphygmomanometer (Omron HEM-629). The mean values were used in the analyses.
• HDL cholesterol was measured using an ultrasensitive direct method, with a Selectra 2 analyzer (Merck).
• Triglyceride was assessed with a colorimetric enzymatic method.
• Random blood glucose: was assessed from fingertip blood, at the time of collecting the blood samples, using a portable glucose meter (Accu-Check Advantage – Roche).
• C-reactive protein was measured using an Immulite chemiluminescent immunoassay (Siemens). Because the lower detection limit was 0.1 mg/L, measures below that value were converted to 0.05 mg/L. Subjects with CRP >10 mg/L were excluded from the analyses involving CRP, as were pregnant women and those using oral contraceptives.

With respect to the exposure to maternal smoking in the pregnancy, during the interview at the hospital, the mothers were asked if they had smoked during the pregnancy. Those mothers who reported any intake during pregnancy were considered as smokers. Information on the duration of smoking during the pregnancy (mother stopped smoking during pregnancy or not) and the number of cigarette smoked was also gathered.

Because glucose levels were related to fasting time, glucose estimates were corrected for the time elapsed since the last meal. [10] A linear regression estimated the mean change in random glucose according to fasting time. Thereafter, we corrected the glucose estimates.

Information on confounding variables was collected in the early phases of the study. These included monthly family income, maternal education, household assets index (obtained through factor analysis and based on the ownership of household goods) and maternal skin color.

Triglycerides and CRP values (mg/L) were natural log-transformed (lnmg/L) for greater symmetry prior to undertaking statistical analyses and are presented in the text as the geometric mean. In the crude analysis, means were compared using analysis of variance. Adjusted analyses, controlling for the above listed confounders, were carried out using linear regression analysis. Statistical comparisons between groups were based on tests of heterogeneity and linear trend in the case of ordinal variables, and the one with the lower *P*-value was presented.

For those risk factors that were associated to exposure to maternal smoking in the pregnancy, we also adjusted for possible mediators. The adjustment for mediators followed a stepwise model, initially perinatal factors (birthweight) were included in the regression models. Then, adult behavioral factors were also included (leisure-time physical activity, dietary pattern, intake of fat and fiber, and tobacco smoking) and finally estimates were adjusted for adult anthropometry (body mass index and waist circumference). Information about leisure-time physical activity was collected using the International Physical Activity Questionnaire. Those with weekly physical activity below 150 min were considered as sedentary at leisure-time. With respect to dietary patterns, information on diet in the last 12 months was evaluated using a Food Frequency Questionnaire. And, a principal component analysis identified three main dietary patterns (common Brazilian, processed food and prudent type).

The study was approved by the Ethical Review Board of the Faculty of Medicine of the Federal University of Pelotas, and written informed consent was obtained from participating subjects.

## 3 Results

In the 2004–2005 follow-up visit, 4297 subjects were interviewed. Added to the 282 known to have died, they represented a follow-up rate of 77.4%. Follow-up rates were independent of birthweight, sex and maternal skin color. On the other hand, children born at either the upper or lower end of family income distribution and those whose mother had 12 or more years of schooling were less likely to be traced in adulthood [11].

Table 1 shows that 14.4% of the subjects evaluated at 23 years were born small for gestational age, whereas 5.3% were preterm. And about one of every three subjects was exposed to maternal smoking during pregnancy. At 23 years, mean systolic blood pressure was 117.5 mmHg and HDL cholesterol was 55.5 mg/dL.

To help interpret the results of the adjusted analyses, it is important to understand the associations between maternal smoking and confounding/mediating factors, and among the latter and the outcomes under study. Maternal smoking was inversely associated with family income, maternal education, and household asset index (Web Table 1). In terms of associations between maternal smoking and potential mediating factors, maternal smoking in the pregnancy was positively associated with tobacco smoking in early adulthood, whereas fat intake was slightly lower among those subjects whose mother smoked in the pregnancy (Web Table 2). Prevalence of tobacco smoking in early adulthood is inversely associated with socioeconomic status (Web Table 3). Web Table 4 presents the distribution of the metabolic risk factors of cardiovascular disease according to the confounding variables. Triglycerides, HDL cholesterol and C-reactive protein were positively associated with socioeconomic variables, whereas blood pressure was related to maternal skin color.

In the crude analyses, the only outcome associated with maternal smoking was HDL cholesterol in females, but not in males (*P* value for interaction with sex equal to 0.009). Women born to smoking mothers had lower HDL levels (Table 2).

Table 3 shows that even after controlling for possible confounding variables HDL cholesterol levels were still lower among female offspring of smokers. Independent of gender, C-reactive protein was lower among those subjects whose mother smoked 15 or more cigarettes/day in the pregnancy. The confidence intervals, however, included the null value, and there was no evidence of an effect among light smokers, nor was CRP related to duration of exposure to smoking during pregnancy.

Analyses adjusted for potential mediating factors were carried out for HDL cholesterol among females, the only association that persisted in the confounder adjusted analyses. After adjustment for lifestyle variables in early adulthood (leisure time physical activity, intake of fat, diet pattern and tobacco smoking), birthweight, and waist circumference, the difference in HDL levels between female offspring of smokers and non-smokers reduced from −2.10 mg/dL (95% confidence interval: −3.39; −0.80) to −1.03 mg/dL (−2.35; 0.30) (Table 4).

## 4 Discussion

In this cohort that has been followed up since birth, the only evidence that maternal smoking is associated with the development of metabolic cardiovascular risk factors in early adulthood was the observation that HDL cholesterol was lower among offspring of smokers. Because of the large number of comparisons included in the analyses, the possibility of a chance finding cannot be discarded. Also, the disappearance of the association after adjustment for risk factors measured in young adults suggests that – if true – the observed association was mediated through contemporary variables, as reported by Power et al. for the 1958 British cohort [8].

In the 2004–2005 visit, we were not able to locate 22.6% of the subjects, as previous cited follow-up rate was not related to sex, maternal skin color and birthweight. However, the attrition rate was lower among subjects in middle-income group. Because maternal smoking during pregnancy was not related to attrition rate, selection bias is unlikely to have affected our results. Another possible limitation of the analyses is the young age of subjects, before the peak age range for cardiovascular diseases. Nevertheless, metabolic risk factors, such as blood lipids measured in young adults, are related to a higher risk of cardiovascular disease in midlife. One cannot rule out the possibility that the effects of maternal smoking on precursors of chronic diseases will only become apparent at later ages.

Information on maternal smoking during pregnancy was collected just after delivery, minimizing misclassification. Furthermore, information on confounding variables was collected prospectively, using standardized questionnaires and trained interviewers. One limitation was that lipids and glucose levels were measured from non-fasting blood, as obtaining fasting samples in such a large cohort presented major logistic difficulties. Whereas HDL cholesterol is not affected by fasting status [12], triglycerides vary according to time since last meal and time of day. [13] In the literature, fasting triglycerides are preferred, mainly due to lower variability in the measurement and the need to estimate LDL with Friedwald equation, using HDL cholesterol and triglyceride levels. [14] However, epidemiological studies have reported that non-fasting triglycerides predict cardiovascular risk better than fasting levels. [15–18] Therefore, the use of non-fasting samples is acceptable in this analysis on the programming of metabolic cardiovascular risk factors by maternal smoking in pregnancy. With respect to blood glucose, time since last meal was not related to maternal smoking during pregnancy, and, therefore, the use of non-fasting samples introduced a non-differential classification bias. Because, no pattern that would suggest an association between maternal smoking during pregnancy and blood glucose levels in adulthood was observed, this classification bias cannot be considered as responsible for the lack of association between maternal smoking and blood glucose levels.

As mentioned, the evidence on the long-term effect of maternal smoking during pregnancy on metabolic cardiovascular risk factors is controversial. A meta-analysis observed a small increase in blood pressure among offspring of smokers, [5] but the findings of similar effects of both paternal and maternal smoking on blood pressure [7] and the strong reduction in the magnitude of the association between maternal smoking and offspring cardiovascular risk factors in adulthood after adjustment for lifestyle variables [8] raised the issue that the observed long-term effect was a marker of familial factors that are related to lifestyle risk factors of cardiovascular disease.

Indeed, the prevalence of lifestyle cardiovascular risk factors tends to be higher among offspring of smoking parents. A literature review reported that parental smoking was positively associated with smoking in adolescence, [19] whereas another study observed that maternal smoking in pregnancy was related to smoking in girls but not in boys. [20] With respect to diet, maternal smoking has been related to breastfeeding duration [21], as well as to diet quality and physical activity in childhood and adolescence. [22–24] Nevertheless, existing data suggest that such association is not due to prenatal exposure to smoking, but more likely due to intergenerational transmission of health behaviors.

To conclude there are little evidence that maternal smoking during pregnancy program offspring metabolic cardiovascular risk factors, and reported associations are likely due to postnatal exposure to lifestyle patterns.

## Funding

This work was supported by the Wellcome Trusts initiative entitled Major Awards for Latin America on Health Consequences of Population Change and Rio Grande do Sul State Research Support Foundation (FAPERGS). Earlier phases of the 1982 cohort study were funded by the International Development Research Center (Canada), the World Health Organization (Department of Child and Adolescent Health and Development, and Human Reproduction Programme), the Overseas Development Administration (United Kingdom), the United Nations Development Fund for Women, the National Program for Centers of Excellence (Brazil), the National Research Council (Brazil) and the Ministry of Health (Brazil).

## Figures and Tables

**Table 1:** Distribution of sample studied at 23 years of age, according to key characteristics.

Sample characteristics	*N*	Mean (SD)	Prevalence (%)
At birth			
Birthweight (g)	4295	3223 (517)	
Preterm birth	3442		5.3
Small-for-gestational age	3441		14.4
Maternal smoking in the pregnancy			
No	2765		64.5
Stopped smoking during pregnancy	348		8.1
Smoked during whole pregnancy	1176		27.4
At 23 years			
Triglycerides (mg/dL)^a^	3663	91.4 (1.71)	
HDL cholesterol (mg/dL)	3824	55.5 (13.0)	
Non-fasting glucose (mg/dL)^b^	3706	93.8 (16.1)	
Mean systolic blood pressure (mmHg)	4291	117.5 (15.0)	
Mean diastolic blood pressure (mmHg)	4291	73.6 (11.5)	
C-reactive protein^a^	3091	0.97 (3.32)	

a Geometric mean.

b Corrected for fasting time.

**Table 2:** Metabolic cardiovascular risk factors according to maternal smoking during pregnancy. Unadjusted analyses.

	Mean triglycerides in mg/dL (SD)^a^	Mean HDL cholesterol in mg/dL (SD)	Mean non-fasting glucose (SD) in mg/dL	Mean systolic blood pressure in mmHg (SD)	Mean diastolic blood pressure in mmHg (SD)	Mean C-reactive protein (SD)^a^ in mg/L
Male						
Maternal smoking during pregnancy	*P* = 0.75	*P* = 0.19	*P* = 0.72	*P* = 0.45	*P* = 0.43	*P* = 0.74
No	97.6 (1.79)	51.8 (11.2)	96.2 (16.4)	123.7 (14.3)	75.8 (11.6)	0.79 (3.18)
Yes	96.8 (1.76)	51.1 (11.2)	96.5 (17.2)	123.2 (14.4)	75.4 (11.8)	0.77 (3.16)
Maternal smoking during pregnancy	*P* = 0.07^b^	*P* = 0.31^b^	*P* = 0.45^b^	*P* = 0.06^b^	*P* = 0.34^b^	*P* = 0.21^b^
Non smokers	97.6 (1.79)	51.8 (11.2)	96.2 (16.4)	123.7 (14.3)	75.8 (11.6)	0.79 (3.18)
<15 cig/day	99.7 (1.76)	50.9 (11.3)	96.9 (17.7)	122.5 (14.6)	75.1 (11.7)	0.81 (3.14)
≥15 cig/day	89.0 (1.73)	51.7 (11.0)	95.1 (15.4)	125.1 (13.8)	76.3 (12.1)	0.68 (3.19)
Maternal smoking during pregnancy	*P* = 0.80^b^	*P* = 0.22^b^	*P* = 0.86^b^	*P* = 0.75^b^	*P* = 0.54^b^	*P* = 0.14^b^
Non smokers	97.6 (1.79)	51.8 (11.2)	96.2 (16.4)	123.7 (14.3)	75.8 (11.6)	0.79 (3.18)
Stopped smoking during pregnancy	99.1 (1.72)	52.0 (11.5)	96.0 (14.7)	123.1 (15.2)	76.0 (12.1)	0.91 (3.10) 0.74 (3.17)
Smoked during the whole pregnancy	96.1 (1.77)	50.8 (11.1)	96.6 (17.8)	123.2 (14.2)	75.3 (11.7)	
Female						
Maternal smoking during pregnancy	*P* = 0.33	*P* < 0.001	*P* = 0.23	*P* = 0.99	*P* = 0.39	*P* = 0.41
No	86.6 (1.62)	60.4 (13.6)	91.1 (14.7)	111.1 (12.8)	71.4 (10.4)	1.34 (3.34)
Yes	84.7 (1.61)	57.5 (13.1)	92.0 (15.7)	111.1 (13.4)	71.0 (11.4)	1.26 (3.31)
Maternal smoking during pregnancy	*P* = 0.49^b^	*P* < 0.001^c^	*P* = 0.11^c^	*P* = 0.98^b^	*P* = 0.31^c^	*P* = 0.29^c^
Non smokers	86.6 (1.62)	60.4 (13.6)	91.1 (14.7)	111.1 (12.8)	71.4 (10.4)	1.34 (3.34)
<15 cig/day	84.1 (1.60)	57.7 (13.3)	91.6 (15.5)	111.2 (13.9)	71.1 (11.6)	1.29 (3.26)
≥15 cig/day	86.8 (1.64)	57.1 (12.4)	93.3 (16.4)	110.9 (11.6)	70.6 (10.4)	1.16 (3.51)
Maternal smoking during pregnancy	*P* = 0.52^b^	*P* < 0.001^c^	*P* = 0.47^b^	*P* = 0.39^b^	*P* = 0.19^b^	*P* = 0.55^b^
Non smokers	86.6 (1.62)	60.4 (13.6)	91.1 (14.7)	111.1 (12.8)	71.4 (10.4)	1.34 (3.34)
Stopped smoking during pregnancy	83.0 (1.58)	59.2 (13.5)	92.2 (16.0)	112.3 (13.7)	72.2 (11.7)	1.17 (3.33)
Smoked during the whole pregnancy	85.2 (1.62)	57.1 (12.9)	91.9 (15.6)	110.8 (13.3)	70.7 (11.3)	1.29 (3.31)

a Geometric mean.

b Test for heterogeneity.

c Test for linear trend.

**Table 3:** Adjusted regression coefficients for metabolic cardiovascular risk factors at 23 years according to maternal smoking in the pregnancy.

	Adjusted regression coefficient (95% confidence interval)^c^					
	Triglycerides in mg/dL	Mean HDL cholesterol in mg/dL	Non-fasting glucose in mg/dL	Mean systolic blood pressure in mmHg	Mean diastolic blood pressure in mmHg	C-reactive protein in mg/L
Male						
Maternal smoking during pregnancy	*P* = 0.99	*P* = 0.57	*P* = 0.86	*P* = 0.84	*P* = 0 0.52	*P* = 0.95
No	0.0 (reference)	0.0 (reference)	0.0 (reference)	0.0 (reference)	0.0 (reference)	0.0 (reference)
Yes	0.00 (−0.06; 0.06)	−0.32 (−1.43; 0.79)	0.15 (−1.52; 1.82)	−0.14 (−1.47; 1.20)	−0.36 (−1.44; 0.73)	0.00 (−0.11; 0.12)
Maternal smoking during pregnancy	*P* = 0.03^b^	*P* = 0.68^b^	*P* = 0.74^b^	*P* = 0.16^b^	*P* = 0.60^b^	*P* = 0.10^b^
Non smokers	0.0 (reference)	0.0 (reference)	0.0 (reference)	0.0 (reference)	Reference	0.0 (reference)
<15 cig/day	0.03 (−0.03; 0.10)	−0.50 (−1.73; 0.72)	0.44 (−1.40; 2.29)	−0.74 (−2.22; 0.73)	−0.56 (−1.76; 0.64)	0.06 (−0.07; 0.19)
≥15 cig/day	−0.10 (−0.19; −0.01)	0.19 (−1.67; 2.05)	−0.73 (−3.57; 2.10)	1.58 (−0.64; 3.81)	0.23 (−1.59; 2.04)	−0.17 (−0.37; 0.03)
Maternal smoking during pregnancy	*P* = 0.99^b^	*P* = 0.30^b^	*P* = 0.85^a^	*P* = 0.93^b^	*P* = 0.62^b^	*P* = 0.24^b^
Non smokers	0.0 (reference)	0.0 (reference)	0.0 (reference)	0.0 (reference)	Reference	0.0 (reference)
Stopped smoking during pregnancy	0.01 (−0.09; 0.10)	0.85 (−1.10; 2.80)	0.03 (−2.93; 2.99)	−0.47 (−2.82; 1.88)	0.24 (−1.67; 2.15)	0.15 (−0.06; 0.35)
Smoked during the whole pregnancy	0.00 (−0.06; 0.06)	−0.69 (−1.91; 0.53)	0.18 (−1.65; 2.01)	−0.04 (−1.49; 1.42)	−0.54 (−1.72; 0.65)	−0.04 (−0.17; 0.09)
Female						
Maternal smoking during pregnancy	*P* = 0.57	*P* = 0.002	*P* = 0.60	*P* = 0.99	*P* = 0.57	*P* = 0.35
No	0.0 (reference)	0.0 (reference)	0.0 (reference)	0.0 (reference)	0.0 (reference)	0.0 (reference)
Yes	−0.01 (−0.06; 0.03)	−2.10 (−3.39; −0.80)	0.40 (−1.12; 1.92)	0.00 (−1.23; 1.24)	−0.29 (−1.31; 0.72)	−0.07 (−0.22; 0.08)
Maternal smoking during pregnancy	*P* = 0.62^b^	*P* = 0.002^a^	*P* = 0.36^a^	*P* = 0.99^b^	*P* = 0.50^a^	*P* = 0.20^a^
Non smokers	0.0 (reference)	0.0 (reference)	0.0 (reference)	0.0 (reference)	0.0 (reference)	0.0 (reference)
<15 cig/day	−0.02 (−0.07; 0.03)	−1.92 (−3.33; −0.51)	0.03 (−1.61; 1.68)	−0.02 (−1.35; 1.31)	−0.21 (−1.31; 0.90)	−0.04 (−0.20; 0.12)
≥15 cig/day	0.02 (−0.07; 0.10)	−2.73 (−5.10; −0.37)	1.73 (−1.03; 4.49)	0.09 (−2.18; 2.36)	−0.62 (−2.50; 1.26)	−0.20 (−0.47; 0.07)
Maternal smoking during pregnancy	*P* = 0.84^b^	*P* < 0.001^a^	*P* = 0.83^b^	*P* = 0.19^b^	*P* = 0.05^b^	*P* = 0.64^b^
Non smokers	0.0 (reference)	0.0 (reference)	0.0 (reference)	0.0 (reference)	0.0 (reference)	0.0 (reference)
Stopped smoking during pregnancy	−0.02 (−0.10; 0.06)	−0.50 (−2.81; 1.82)	0.79 (−1.92; 3.49)	1.67 (−0.51; 3.85)	1.50 (−0.30; 3.31)	−0.08 (−0.35; 0.18)
Smoked during the whole pregnancy	−0.01 (−0.06; 0.04)	−2.57 (−3.98; −1.15)	0.29 (−1.36; 1.94)	−0.50 (−1.84; 0.85)	−0.84 (−1.95; 0.28)	−0.07 (−0.23; 0.09)

a Test for linear trend.

b Test for heterogeneity.

c Adjusted for: family income at birth, maternal schooling, skin color and household asset index.

**Table 4:** Adjusted regression coefficients for HDL cholesterol at 23 years according to maternal smoking in the pregnancy, for females.

	Adjusted regression coefficient (HDL cholesterol in mg/dL)			
	Adjusted for confounding	1 + birth weight	2 + lifestyle 23 years	3 + Body mass index and waist circumference
Maternal smoking in the pregnancy	*P* = 0.002	*P* = 0.02	*P* = 0.08	*P* = 0.13
No	0.0 (reference)	0.0 (reference)	0.0 (reference)	0.0 (reference)
Yes	−2.10 (−3.39; −0.80)	−1.62 (−2.94; −0.31)	−1.16 (−2.47; 0.15)	−1.03 (−2.35; 0.30)
Maternal smoking in the pregnancy	*P* = 0.002*	*P* = 0.02*	*P* = 0.06*	*P* = 0.13*
No	0.0 (reference)	0.0 (reference)	0.0 (reference)	0.0 (reference)
<15 cig/day	−1.92 (−3.33; −0.51)	−1.53 (−2.95; −0.12)	−0.99 (−2.39; 0.42)	−0.95 (−2.38; 0.47)
≥15 cig/day	−2.73 (−5.10; −0.37)	−1.97 (−4.35; 0.42)	−1.83 (−4.23; 0.57)	−1.31 (−3.75; 1.13)
Maternal smoking in the pregnancy	*P* < 0.001*	*P* = 0.007*	*P* = 0.06*	*P* = 0.12*
No	0.0 (reference)	0.0 (reference)	0.0 (reference)	0.0 (reference)
Stopped smoking during pregnancy	−0.50 (−2.80; 1.83)	−0.30 (−2.61; 2.01)	−0.40 (−2.70; 1.89)	−0.66 (−2.98; 1.67)
Smoked during the whole pregnancy	−2.57 (−3.98; −1.15)	−2.02 (−3.46; −0.59)	−1.39 (−2.82; 0.05)	−1.14 (−2.60; 0.32)

1. Adjusted for family income at birth, maternal schooling, skin color and household asset index. 2. Adjusted for family income at birth, maternal schooling, skin color, household asset index and birthweight. 3. Adjusted for family income at birth, maternal schooling, skin color, household asset index, birthweight and lifestyle variables at 23 years.

* Test for linear trend.
