# Supplementary material for: Maternal smoking during pregnancy and risk factors for cardiovascular disease in adulthood
Source: Atherosclerosis. 2011 Dec;219(2):815–20. doi: 10.1016/j.atherosclerosis.2011.08.018 (PMC3234339; doi:10.1016/j.atherosclerosis.2011.08.018)
Supplement: Supplementary file 1 [file mmc1.doc]

Web Table 1. Maternal smoking according to confounding variables

|  | Prevalence of maternal smoking in the pregnancy (%) |
| --- | --- |
| Family income at birth in minimum wages  ≤ 1  1.1 – 3  3.1 – 6  6.1 – 10  > 10 | P < 0.001 @  43.9  36.3  32.5  20.2  25.1 |
| Maternal schooling at birth (in years)  ≤ 4  5 – 8  9 – 11  ≥ 12 | P < 0.001 @  37.6  38.4  32.6  24.9 |
| Maternal skin color  White  Black or mixed | P < 0.001  34.5  40.7 |
| Household score asset index in quintiles  1^st^  2^nd^  3^rd^  4^th^  5^th^ | P < 0.001 @  44.4  37.5  32.1  31.1  29.7 |

@ test for heterogeneity

Web Table 2. Mediating Factors according to maternal smoking

|  | Prevalence ratio or mean difference (95% confidence interval) | | | | | |
| --- | --- | --- | --- | --- | --- | --- |
|  | Sedentary at leisure time | Score of prudent type dietary pattern | Score of processed food dietary pattern | Low fiber diet | High or very high fat intake | Smoking at 23 years |
| Maternal smoking during pregnancy  Yes  No | p=0.06  0.95 (0.91; 1.01)  Reference (1) | p = 0.67  0.02 (-0.07; 0.11)  Reference (0) | p=0.55  -0.03 (-0.13; 0.07)  Reference (0) | p=0.36  1.03 (0.97; 1.09)  Reference (1) | p = 0.04  0.95 (0.90; 0.99)  Reference (1) | P = 0.04  1.17 (1.01; 1.35)  Reference (1) |

Web Table 3. Tobacco smoking at 23 years according to confounding variables

|  | Prevalence of tobacco smoking at 23 years (%) |
| --- | --- |
| Family income at birth in minimum wages  ≤ 1  1.1 – 3  3.1 – 6  6.1 – 10  > 10 | P < 0.001 *  32.2  26.7  21.9  18.7  14.3 |
| Maternal schooling at birth (in years)  ≤ 4  5 – 8  9 – 11  ≥ 12 | P < 0.001 *  31.0  25.9  18.8  17.5 |
| Maternal skin color  White  Black or mixed | P < 0.001  24.6  30.7 |
| Household score asset index in quintiles  1^st^  2^nd^  3^rd^  4^th^  5^th^ | P < 0.001 @  34.7  24.9  24.0  21.0  19.9 |

@ test for heterogeneity

* test for linear trend

Web Table 4. Metabolic cardiovascular risk factors according to confounding variables.

|  | Mean triglycerides in mg/dL (SD) # | Mean HDL cholesterol in mg/dL (SD) | Mean Non-fasting glucose (SD) in mg/dL | Mean systolic blood pressure in mmHg (SD) | Mean diastolic blood pressure in mmHg (SD) | Mean C-reactive protein (SD) # in mg/l |
| --- | --- | --- | --- | --- | --- | --- |
| Family income at birth in minimum wages  ≤ 1  1.1 – 3  3.1 – 6  6.1 – 10  > 10 | P < 0.001 @  85.2 (1.65)  91.5 (1.71)  95.9 (1.75)  95.6 (1.74)  96.3 (1.64) | P < 0.001 *  54.4 (12.4)  54.6 (12.7)  56.6 (13.6)  59.2 (13.0)  60.9 (13.6) | P = 0.36 *  93.8 (16.7)  93.5 (15.1)  94.7 (17.9)  95.2 (16.8)  93.5 (15.3) | P = 0.93 @  117.3 (15.4)  117.7 (15.1)  117.4 (14.6)  117.6 (15.1)  116.9 (14.9) | P = 0.57 @  73.7 (12.2)  73.4 (11.6)  73.8 (10.6)  74.5 (11.2)  73.2 (11.0) | P = 0.04 @  0.86 (3.43)  0.97 (3.35)  1.06 (3.33)  0.95 (2.93)  1.05 (2.97) |
| Maternal schooling at birth (in years)  ≤ 4  5 – 8  9 – 11  ≥ 12 | P < 0.001 *  88.7 (1.70)  90.4 (1.70)  95.5 (1.72)  99.2 (1.73) | P < 0.001 *  54.2 (12.4)  55.1 (12.8)  56.3 (13.8)  59.5 (13.5) | P = 0.29 @  93.3 (15.4)  94.3 (16.8)  94.1 (15.9)  93.3 (15.2) | P = 0.78 @  117.4 (15.5)  117.3 (14.9)  118.0 (14.6)  117.9 (14.8) | P = 0.28 @  73.3 (12.0)  73.4 (11.3)  74.2 (11.2)  74.2 (10.7) | P = 0.95 @  0.98 (3.33)  0.96 (3.44)  0.99 (3.15)  0.94 (3.12) |
| Maternal skin color  White  Black or mixed | P < 0.001  95.0 (1.70)  77.0 (1.69) | P = 0.15  55.3 (13.1)  56.1 (12.6) | P = 0.11  94.0 (16.0)  92.9 (16.5) | P = 0.03  117.3 (14.9)  118.6 (15.8) | P = 0.32  73.5 (11.2)  73.9 (12.6) | P = 0.006  0.99 (3.28)  0.85 (3.51) |
| Household score asset index in quintiles  1^st^  2^nd^  3^rd^  4^th^  5^th^ | P < 0.001 *  86.1 (1.68)  89.3 (1.72)  91.3 (1.70)  95.8 (1.70)  95.8 (1.69) | P < 0.001 *  52.5 (12.0)  54.8 (12.7)  55.9 (13.2)  57.0 (13.8)  57.2 (13.1) | P = 0.38 @  94.3 (15.7)  94.1 (16.8)  94.1 (16.3)  92.4 (15.1)  93.2 (15.2) | P = 0.75 @  117.5 (14.9)  116.9 (15.3)  117.6 (15.1)  117.9 (14.9)  117.2 (14.8) | P = 0.40 @  73.3 (12.2)  72.9 (11.3)  73.8 (11.6)  73.8 (10.6)  73.7 (10.7) | P = 0.02 *  0.86 (3.23)  0.95 (3.42)  0.99 (3.32)  0.99 (3.36)  1.03 (3.17) |

# geometric mean

@ test for heterogeneity

* test for linear trend
